# Three-Dimensional Models of Liver Vessels for Navigation during Laparotomic Attenuation of Intrahepatic Portosystemic Shunt in Dogs

**DOI:** 10.3390/ani13122004

**Published:** 2023-06-15

**Authors:** Jan Frymus, Piotr Trębacz, Aleksandra Kurkowska, Mateusz Pawlik, Anna Barteczko, Michał Barański, Marek Galanty

**Affiliations:** 1 Department of Small Animal Diseases with Clinic, Institute of Veterinary Medicine, Warsaw University of Life Sciences—SGGW, Nowoursynowska 159c, 02-776 Warsaw, Poland; lek.wet.mikhalbaranski@gmail.com (M.B.); marek_galanty@sggw.edu.pl (M.G.); 2 CABIOMEDE Sp. z o. o., Kielce Technology Park, Olszewskiego 6, 25-663 Kielce, Poland; aleksandra.kurkowska@cabiomede.com (A.K.); mateusz.pawlik@cabiomede.com (M.P.); anna.barteczko@cabiomede.com (A.B.)

**Keywords:** portosystemic shunt, attenuation, dogs, 3-dimensional liver models

## Abstract

**Simple Summary:**

Portosystemic shunt (PSS) is a common abnormality in dogs where through an aberrant vessel, blood from the intestines bypasses the liver. Lack of hepatic detoxication can lead to a fatal outcome. The treatment of choice is a surgical closure of the shunt. In the case of the intrahepatic location of the shunt, its identification is often difficult and requires traumatic preparation of the liver, which influences the postoperative prognosis. Therefore, in order to reduce liver trauma, we printed 3-dimensional (3D) individual patient liver models, scaled 1:1, and used them for planning the surgery and as a guide during intraoperative identification of the shunt. Here, we present the application of this method in four dogs with intrahepatic PSS. The advantages of the 3D technology are simple and precise planning of the surgery, fast intraoperative identification of the shunt, and low invasive dissection of the liver parenchyma. We conclude that 3D technology can potentially raise the recovery rate.

**Abstract:**

Laparotomic attenuation of an intrahepatic portosystemic shunt (IHPSS) is more difficult than an extrahepatic one, and results in a higher risk of complications because the identification of the aberrant vessel in the liver remains often a challenge. Excessive preparation and traction of the parenchyma results in trauma, bleeding, and prolonged surgery, which is what worsens the prognosis. Therefore, based on computed tomographic angiography, we printed 3-dimensional (3D) individual patient liver models, scaled 1:1, and used them for surgery planning and as a guide during intraoperative identification of the shunt in four dogs with IHPSS. The advantages of the 3D technology are simple and precise planning of the surgery, fast intraoperative identification of the shunt, and low invasive dissection of the liver parenchyma. We conclude that 3D technology can potentially raise the recovery rate. To the best of our knowledge, this was the first application of 3D models in the surgery of canine IHPSS.

## 1. Introduction

Portosystemic shunt (PSS) is a common, usually congenital vascular anomaly in dogs and cats. In such patients, the blood from the intestinal tract and some other organs does not pass through the liver via the portal vein, but partially or completely bypasses the liver through one or more aberrant vessels connecting the portomesenteric system to the systemic venous circulation [1,2]. As a result, toxic substances such as ammonia and others accumulate in the systemic circulation. Though the clinical results of PSS are affected by the degree of blood shunting, they can be various and potentially severe, including not only growth retardation, liver atrophy, and gastrointestinal signs, but also hepatic encephalopathy, ammonium urate uroliths, anemia, and many other abnormalities [2,3,4,5,6].

Plane radiography provides limited information on liver vascular abnormalities in dogs. In contrast, ultrasonography (US) brought significant progress in detecting the shunts, and in addition, is commonly available, and does not require general anesthesia; therefore, it is the most used technique in PSS diagnostics in dogs and cats [2,7]. In the case of intrahepatic PSS (IHPSS), color Doppler US reveals blood flow turbulence in the region of the shunt. Finally, the hepatic division can be determined with US. Nevertheless, computed tomographic angiography (CTA) is more accurate than US in imaging the morphology of the vessel and has become the gold standard for the detection of PSS [2,8,9].

Several surgical procedures such as laparotomy, laparoscopy, and endovascular surgery have been described for the treatment of PSS. Though percutaneous endovascular embolization, being minimally invasive, is considered the gold therapeutic standard [10,11], this procedure is not commonly available. Therefore, still, open surgical techniques are mostly used to attenuate the shunts using different types of constrictors, occluders or cellophane bands [12,13]. Identification of the shunting vessel(s) is crucial during this procedure. Based on previous US examinations, identification is usually not difficult by direct visualization after laparotomy if it is an extrahepatic PSS (EHPSS) in typical localization. In contrast, in the case of IHPSS, this is more difficult as the shunt is located within the liver parenchyma [12,13]. The location and anatomical structure of these aberrant vessels vary between the cases, which underlines the importance of reliable navigation for surgery. In such cases, venoportography or intraoperative US is helpful but ideally is a 3-dimensional (3D) presentation. Even if CTA images significantly improve understanding of the vessel morphology on a case-by-case basis, ultimately, the surgeon can see the anatomy of the intrahepatic vessel(s) projected on a 2-dimensional monitor during the surgery. To face such problems, 3D-printed models produced on the basis of preoperative CT images have been increasingly used in different fields of human and veterinary surgery [14,15,16,17]. Recently, such models of the portal and hepatic vein anatomy have been proposed to support PSS surgery in experimental dogs [18], as well as in two canine patients with natural EHPSS [19]. However, to our best knowledge, no experiences were published of this technology applied in the surgical treatment of canine IHPSS.

Within the last 7 years, 209 dogs with PSS have been referred to our clinic and we operated 200 of them. Based on this background, we aimed to check if 3D 1:1 scaled liver models printed according to CTA individually for each patient, can help in planning and performing the surgery, especially in intraoperative identification of the intrahepatic aberrant vessel as compared with our previous interventions. In this study, we present our experience with the application of 3D modelling in the planning and performing of surgery in four dogs with IHPSS.

## 2. Materials and Methods

### 2.1. Diagnosis of PSS

In all dogs, a suspicion of a PSS was made based on history and symptoms given by the owner, clinical examination, and blood analysis, especially liver function tests. The final diagnosis was made by US examination (Samsung HS50, Ridgefield Park, NJ, USA) and confirmed by CTA (16-slice spiral computed tomography Neuviz 16 Neusoft, Shenyang, China). Liver fragments for histopathological examination were obtained during the surgery to exclude microvascular dysplasia. Hepatic arteriovenous malformation was excluded by CTA.

### 2.2. 3D Modelling

Triangular mesh models obtained by segmenting Digital Imaging and Communications in Medicine (DICOM) CTA images of the patient were preprocessed to prepare files for 3D printing. In the preprocessor software Materialise Magics 25.0, designed to support a range of 3D printing technologies, the mesh was optimized to improve the quality of the model—noise and free triangles were removed. By segmenting the volume of the liver independently from the volume of the shunt, it was possible to create a partial cross-section showing the aberrant vessel within the liver. The machine code to print 3D models was developed in Ultimaker Cura 5.0 slicer software dedicated to fused deposition modelling (FDM) technology. Physical models with a 1:1 scaling between the 3D reconstructed image and the model were made from technical polylactic acid material, using an infill density of 25% and a cross-lattice fill pattern with a single layer thickness of 0.1 mm. Ultimaker 3 (Geldermalsen, The Netherlands) 3D printer was used to produce the models. The last step of the preparation was removing supports and sandblasting with 200 µm glass beads. As the fragments of the model corresponding with the ventral part of the liver parenchyma have been digitally eliminated during printing, the location and morphology of the shunt were exposed and such a model was used for detailed planning of the surgery and for guidance during the operation.

### 2.3. Surgery

**Figure 1:**
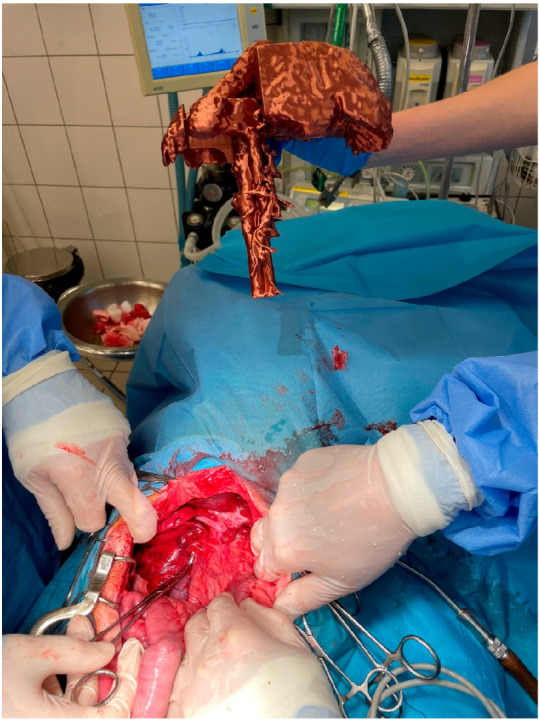
Presentation of the 3D liver model by the assistant during shunt attenuation surgery.

The animals were premedicated i.v. with dexmedetomidine (Dexdomitor, Orion) at a dose of 1–2 µg/kg and fentanyl (Fentadon, Eurovet Animal Health) at a dose of 3–5 µg/kg (in a bolus). Induction was performed i.v. by propofol (Scanofol, ScanVet Poland) at a dose of 1 mg/kg. The anesthesia was maintained by inhalation of isoflurane (Aerrane) in 100% oxygen and i.v. fentanyl constant rate infusion in a dose of 3–5 µg/kg/h. Midline laparotomy in a dorsal position has been performed as described before [20]. In short, the abdominal skin was shaved, and the operating field, including the umbilical area, was prepared according to routine procedures. The abdominal cavity has been opened in the linea alba. During the laparotomy, an assistant presented the 3D model to the surgeons in the position matching the liver of the patient (Figure 1). After identification of the shunt, the pathological vessel was isolated from the surrounding tissue with right-angled dissection forceps (Veterinary Instrumentation, Sheffield, UK). Then, either a single thin cellophane band made from a commercially available cigarette package sterilized with hot steam or an ameroid constrictor (Veterinary Instrumentation) matching the diameter of the vessel was placed around the shunt. If using a cellophane band, the vessel was partially occluded by placing traction on the band. The degree of attenuation was based on visual inspection of the intestines and pancreas during temporary complete ligation for signs of portal hypertension, including increased pulsations of the mesenteric arteries, bowel hypermotility, pancreatic edema, and intestinal and pancreatic cyanosis. Such signs suggest that the dog would not tolerate complete attenuation [21].

## 3. Clinical Cases and Results of the Surgery

Below we show details of the clinical cases and surgery results of 4 client-owned dogs presented to our clinic because of IHPSS.

### 3.1. Case 1

**Figure 2:**
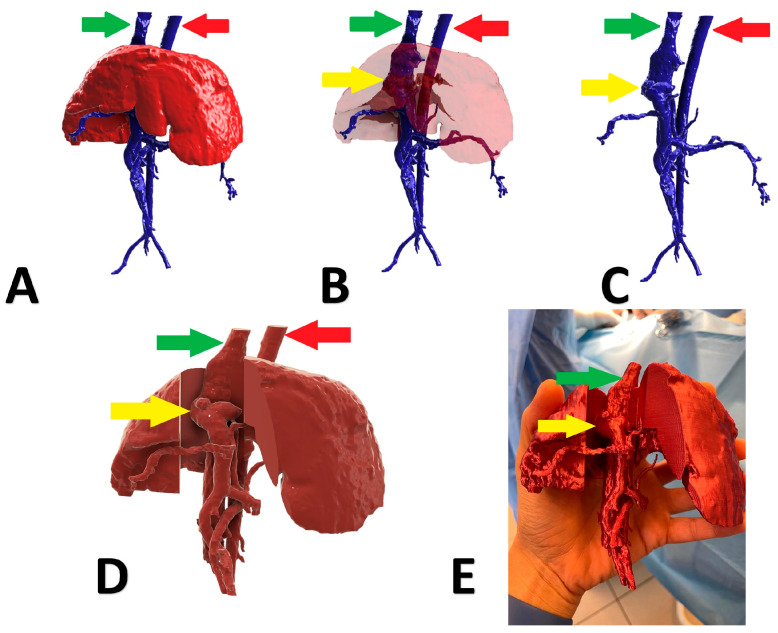
Preoperative stages of the shunt visualization of patient no 1: (**A**) liver and hepatic vessels with invisible shunt based on CTA; (**B**) semitransparent liver with visible shunt based on CTA; (**C**) isolated vessels based on CTA; (**D**) digital visualization of the model with exposed vessels; (**E**) printed 3D model with exposed shunt presented during surgery. Red arrow: aorta; green arrow: caudal vena cava; yellow arrow: shunt; CTA: computed tomographic angiography.

A 6-month-old, male, Nova Scotia Duck Tolling Retriever with right divisional shunt, diameter 10 mm, connecting the right lateral hepatic vein to the caudal vena cava. Attenuation was performed with a cellophane band and confirmed on postoperative US examination. Figure 2 presents the stages of the shunt visualization. The patient showed a good clinical response. At the time of writing, the dog is 6 months post operation. 

### 3.2. Case 2

**Figure 3:**
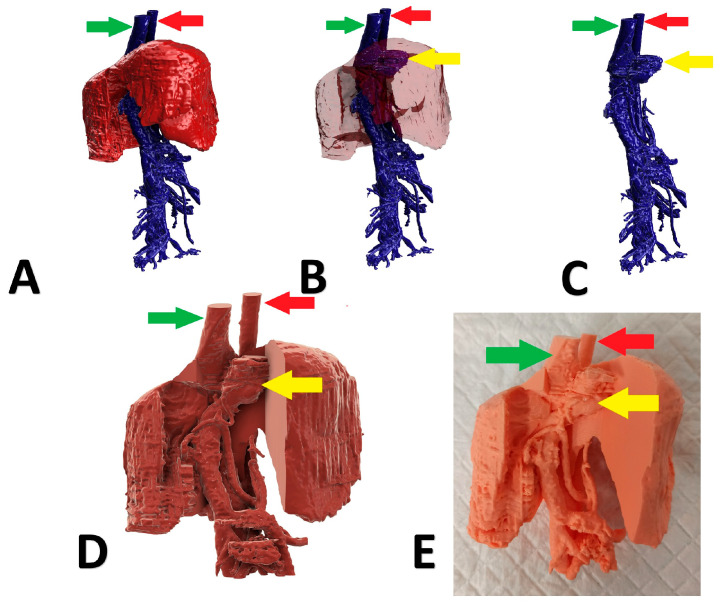
Preoperative stages of the shunt visualization of patient no 2: (**A**) liver and hepatic vessels with invisible shunt based on CTA; (**B**) semitransparent liver with visible shunt based on CTA; (**C**) isolated vessels based on CTA; (**D**) digital visualization of the model with exposed vessels; (**E**) printed 3D model with exposed shunt. Red arrow: aorta; green arrow: caudal vena cava; yellow arrow: shunt; CTA: computed tomographic angiography.

A 6-month-old, female English Cocker Spaniel with a left divisional shunt, diameter 13 mm, connecting the left hepatic vein to the caudal vena cava. Attenuation was performed with a cellophane band and confirmed on postoperative US examination. Figure 3 presents the stages of the shunt visualization. The patient showed a good clinical response. At the time of writing, the dog is 6 months post operation.

### 3.3. Case 3

**Figure 4:**
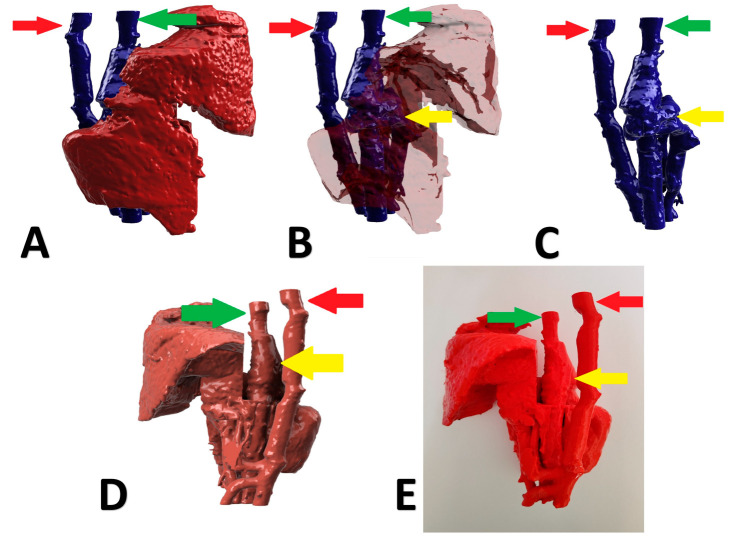
Preoperative stages of the shunt visualization of patient no 3: (**A**) liver and hepatic vessels with invisible shunt based on CTA; (**B**) semitransparent liver with visible shunt based on CTA; (**C**) isolated vessels based on CTA; (**D**): digital visualization of the model with exposed vessels; (**E**): 3D printed model with exposed shunt. Red arrow: aorta; green arrow: caudal vena cava; yellow arrow: shunt; CTA: computed tomographic angiography.

A 5-month-old, male, Polish Lowland Sheepdog with right divisional shunt, diameter 10 mm, connecting the right lateral hepatic vein to the caudal vena cava. Attenuation was performed with an ameroid constrictor (7 mm) and confirmed on postoperative US examination. Figure 4 presents the stages of the shunt visualization. The patient showed a good clinical response. At the time of writing, the dog is 6 months post operation.

### 3.4. Case 4

**Figure 5:**
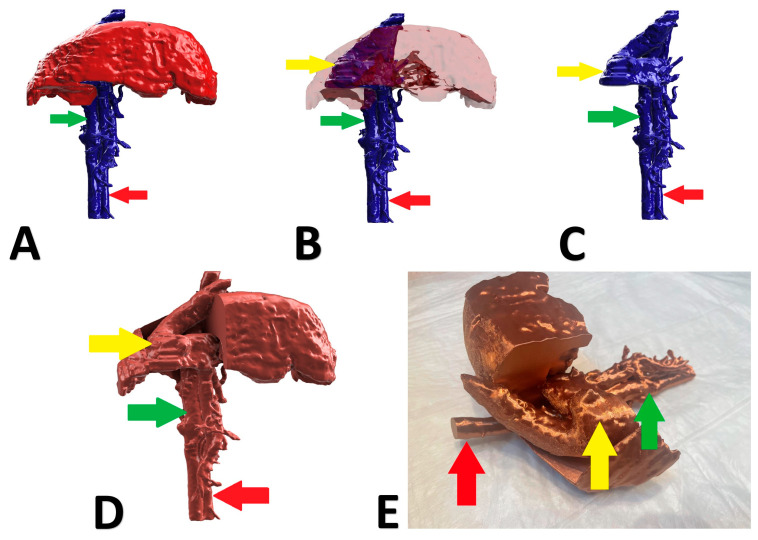
Preoperative stages of the shunt visualization of patient no 4: (**A**) liver and hepatic vessels with invisible shunt based on CTA; (**B**) semitransparent liver with visible shunt based on CTA; (**C**) isolated vessels based on CTA; (**D**) digital visualization of the model with exposed vessels; (**E**) 3D printed model with exposed shunt. Red arrow: aorta; green arrow: caudal vena cava; yellow arrow: shunt; CTA: computed tomographic angiography.

**Figure 6:**
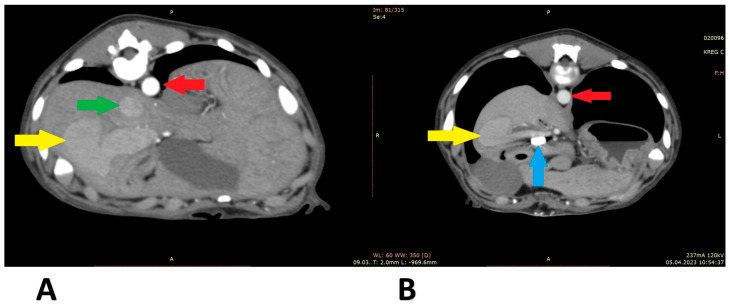
Computed tomographic angiography scans (patient 4): (**A**) before shunt attenuation; (**B**) post attenuation. Red arrow: aorta; green arrow: caudal vena cava; yellow arrow: shunt; blue arrow: vascular clips keeping the cellophane band in position.

A 6-month-old, male, mix-breed with a right divisional shunt, diameter 12 mm, connecting the right lateral hepatic vein to the caudal vena cava. Attenuation was performed with a cellophane band and confirmed on postoperative CTA. Figure 5 shows the stages of the shunt visualization. Figure 6 presents CTA scans of the shunt before and immediately post attenuation. The patient showed a good clinical response. At the time of writing, the dog is 2 months post operation. 

During the surgical interventions, it turned out that all 3D liver models correctly reflected the morphology of the intrahepatic vessels, which helped to find the aberrant vessels. A realistic vision of the anatomical structures scaled 1:1 was superior to plane CT views. All patients improved clinically and during the writing of this manuscript were alive. 

## 4. Discussion

Detailed knowledge of the anatomy of the vascular anomaly in PSS treatment is of great importance for clinical decision making, for planning interventional procedures, and for performing the operation, especially in the case of IHPSS and atypical EHPSS. During surgical treatment of IHPSS, it is usually necessary to incise the liver in order to expose and ligate the aberrant blood vessel. It is important to identify the shunt as quickly as possible because due to dissection of the parenchyma and other manipulations, the liver tissue becomes edematous, crumbly, and discolored by extravasations along with parenchymal bleeding, which quickly makes the visualization of the shunt more difficult. Though the time needed to identify the shunt varies between cases, the possibility to watch the printed liver model scaled 1:1 with an exposed shunt, can make the localization of the aberrant vessel easier. Although 3D models have been predominantly used in small animal orthopedics so far [16,17], the presented cases suggest that also in vascular surgery they can be helpful. A similar opinion has been recently published after the successful application of this technology to support the surgery of two dogs with EHPSS [19]. 

If the amount of blood bypassing the liver in PSS dogs is substantial, the resulting disease is severe; thus, the risk of general anesthesia and laparotomy is also relatively high. Surgery of IHPSS is technically more challenging than of EHPSS due to intraoperative bleeding as a result of liver parenchyma preparation. Therefore, the risk of complications is also higher. The postoperative mortality rate in IHPSS cases is variable depending on the type of occlusion and many other factors and can be as high as 27.3% [22]. The number of successfully treated four IHPSS cases described in the present report is too low to draw general conclusions about postoperative mortality; however, all four improved clinically, which confirms that the shunt was correctly identified and properly attenuated. One could speculate that low traumatic parenchyma preparation due to guided navigation could contribute to a positive clinical outcome. 

The 3D technology can particularly be helpful in atypical cases where a shunt identified on CTA cannot be found during laparotomy. The need for a PSS reoperation is not uncommon in IHPSS dogs [23]. Applying this 3D technology could possibly prevent such problems. Importantly, the time needed to prepare the models was about 7–10 days in our cases, which is acceptable in such a chronic condition like PSS.

Our models were not sterile and thus could be only presented to the surgeons during the operation by an assistant. Some materials such as the rigid acryl resin, are resistant to heat, allowing for sterilization of the model with ethylene oxide gas for use in the operative field [24]. Such a model can be manipulated during the surgery to confirm positional relationships of the vessels and plan the cutting into the liver parenchyma relative to the intrahepatic vessels. Disposable sterile covers such as those available for endoscopic cameras or cables could be a simple alternative.

One limitation of our findings is the lack of postoperative CTA confirming the attenuation of the pathological vessel in all patients. The main reason for this was the attitude of the three other owners. Considering the significant clinical improvement of their dogs, they were reluctant to accept the risk of further anesthesia. On the other hand, in all these patients, postoperative color Doppler US examinations were performed, and attenuation of the shunts was confirmed. In contrast to what we face in some EHPSS cases, this technique is very sensitive to detecting IHPSS. These shunts are usually clearly visible, typically as coiling structures. Thus, the clinical improvement and ultrasound results suggest that in the three cases without postoperative CTA, the shunting vessels were also properly identified and occluded. 

## 5. Conclusions

To the best of our knowledge, the four cases described in this study were the first applications of three-dimensional models in the surgery of canine intrahepatic portosystemic shunts, where the intraoperative identification of the aberrant vessel is usually challenging. The advantages of the 3D technology are simple and precise planning of the surgery, fast intraoperative identification of the shunt, and low invasive dissection of the liver parenchyma. We conclude that 3D technology can potentially raise the recovery rate.

## Data Availability

Not applicable.
